# SHRINKING AND GROWING SOCIAL NETWORKS IN THE SYDNEY MEMORY AND AGEING STUDY

**DOI:** 10.1093/geroni/igz038.161

**Published:** 2019-11-08

**Authors:** Anne-Nicole S Casey, Nicole A Kochan, Perminder Sachdev, Henry Brodaty

**Affiliations:** 1 Centre for Healthy Brain Ageing, University of New South Wales (UNSW) Sydney, Sydney, New South Wales, Australia; 2 Centre for Healthy Brain Ageing, Sydney, New South Wales, Australia; 3 Centre for Healthy Brain Ageing (CHeBA), UNSW Sydney, Sydney, New South Wales, Australia

## Abstract

Gerontological research suggests that shrinking social networks are characteristic of older age. Socioemotional selectivity theory suggests that older adults proactively reduce their networks over time. Core relationship networks consist of friends and family who are contacted at least monthly. Studies of perceived social support indicate that shrinking networks increase risk of isolation, loneliness, and associated ill health effects. Our research aimed to investigate change in size of older adults’ core relationship network over time, and to explore associations between network size and participant characteristics, using data from the first four waves of the Sydney Memory and Ageing Study. Participants completed: assessments of cognition, depression, personality; surveys of health-related quality of life, medical conditions, number of friends and relatives contacted at least monthly. Of 1037 participants aged 70 to 90 years surveyed at baseline 476 provided network data at all four time-points. Just over half of the participants (n=252, 52.9%) had smaller networks at wave 4 compared to baseline; nearly half of participants had stable (n=65, 13.7%) or larger networks (n=159, 33.4%), some doubling in size (n=65, 13.7%). Chi-square test indicated that more males (59.1%) had decreasing networks [X2(1, 476)=2.26, p=.02, phi=.11]. T-tests indicated: group differences in baseline neuroticism [t(276)=2.31, p=.02, Eta2=.02]; no significant difference in age, education, wave 4 global cognition, depression, quality of life, number of medical conditions. Results complement previous literature, yet challenge assumptions that shrinking social networks are a defining characteristic of older age. Future examination of our mental and physical engagement data may elucidate these differences.

